# Urgent Repair of a 17.3 cm Inflammatory Abdominal Aortic Aneurysm

**DOI:** 10.7759/cureus.19248

**Published:** 2021-11-04

**Authors:** Simone H Mangan, Ramesh Velu

**Affiliations:** 1 General Surgery, Mackay Base Hospital, Mackay, AUS; 2 Vascular Surgery, The Townsville University Hospital, Townsville, AUS

**Keywords:** rural emergency medicine, open repair of aneurysms, emergency surgery, vascular surgery, surveillance, inflammatory abdominal aortic aneurysm

## Abstract

We describe a case of delayed presentation of a very large infra-renal inflammatory abdominal aortic aneurysm. This case highlights the importance of early detection and surveillance of aneurysms in rural communities. Definitive management of symptomatic aneurysms is time critical, and any delay such as for the transfer of patients from a rural site can impact patient survival. We present an example of a rare variant of abdominal aortic aneurysm.

## Introduction

Abdominal aortic aneurysms (AAAs) are a focal dilation of the aorta and defined as an aortic diameter at least one and one-half times the normal diameter at the level of the renal arteries. A segment of an aorta greater than 3.0 cm is considered an AAA. The pathogenesis of aneurysms is not fully understood; however, atherosclerosis and inflammation play a role [1]. Associated risk factors include male gender, age over 60 years, Caucasians, smokers, atherosclerosis, hypertension, and a positive family history [2,3]. Interestingly, diabetes is a protective factor [4,5]. Ruptured AAAs are the 13th leading cause of death in the United States [6]. There are a number of factors that determine the risk of rupture. An aneurysm that is greater than 5.0 cm has a five-year rupture rate of 25-40% compared to 1-7% for aneurysms 4-5 cm in diameter [7,8]. Aneurysms that expand rapidly with an increase in diameter of 0.5 cm or more in six months are at a higher risk of rupture due to the presence of increased wall stress [7,8]. The case presented here had an annual risk of rupture of 30-50%.

## Case presentation

**Figure 1:**
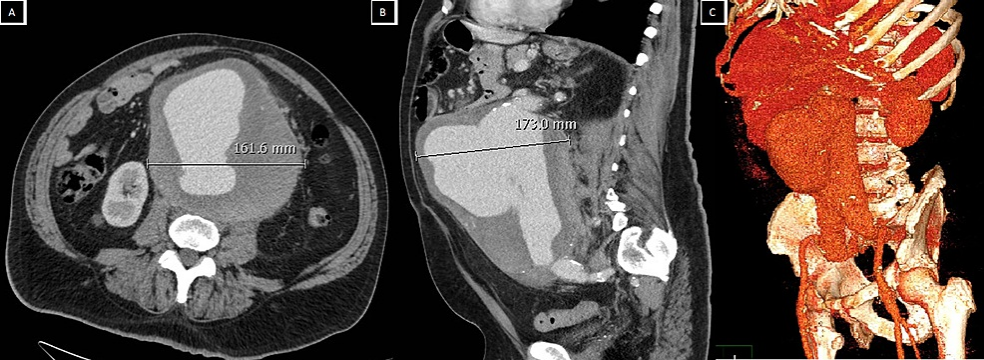
CT-angiogram demonstrating the infra-renal abdominal aortic aneurysm prior to surgery in axial (A), sagittal (B), and reconstruction (left oblique) (C) views.

A 67-year-old Caucasian male presented to his GP in a rural town 600 km from the nearest tertiary hospital, with a two-week history of intermittent peri-umbilical abdominal pain, lower back pain, and an obvious pulsatile mass. He had been aware of the mass for several years; however, he had never had it investigated. An aortic CT-angiogram revealed an infra-renal AAA. It was 17.3 cm in diameter and 22 cm in length (Figure 1) and had no extravasation of contrast or retroperitoneal blood to indicate a rupture. His risk factors for an inflammatory AAA included being male, obese (body mass index [BMI] 36.8), ex-smoker, and previous hypertension, which had resolved five years prior, following 20 kg of weight loss. The patient was not on any regular medications and reported an active lifestyle.

**Figure 2:**
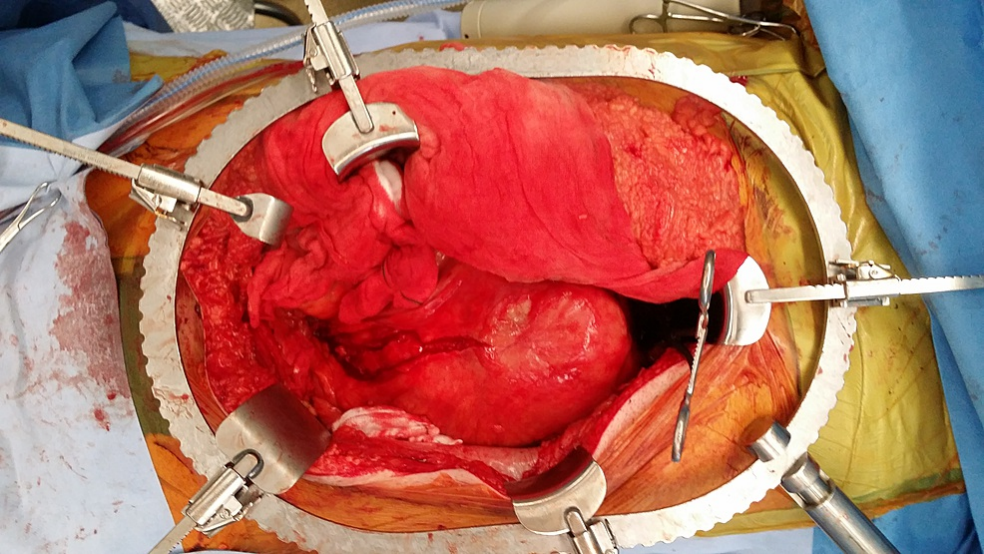
Intra-operative photograph showing the large abdominal aortic aneurysm before the sac was opened.

The patient was urgently transferred to a tertiary hospital where he underwent an open repair under general anesthesia via a midline laparotomy. A large pulsatile inflammatory infra-renal AAA extending to the right common iliac artery was identified (Figure 2).

**Figure 3:**
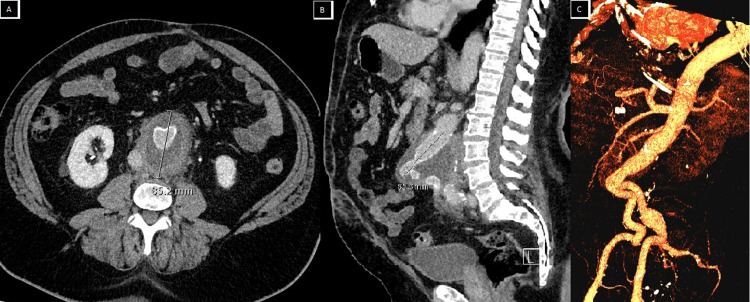
CT-angiogram following open repair in axial (A), sagittal (B), and reconstruction (left oblique) (C) views.

It was adherent to the retroperitoneum. After infra-renal cross-clamping, the iliac arteries were controlled with Fogarty catheters. The aneurysm was opened and a large thrombus was removed. A 22/11 polytetrafluoroethylene graft was anastomosed to the neck and to the right external iliac and left common iliac artery. The right internal iliac artery was ligated. Excess aneurysm wall was removed and the sac was closed. The tissue was unfortunately not sent for histology. The patient had an estimated blood loss of 6000 mL and required six units packed red blood cells, 300 mL cryoprecipitate, 2400 mL from the Cell Saver transfusion, 1500 mL albumin, eight units fresh frozen plasma, and 6000 mL crystalloid. He was extubated the following day. He developed moderate renal impairment but otherwise recovered well. The patient was discharged home eight days after surgery. He had his routine post-operative review at six weeks, followed by regular reviews at 3, 6, 9, and 12 months with a combination of CT-angiograms and Doppler ultrasounds. Follow-up imaging showed the graft was in situ with an interval reduction in the size of the aneurysmal sac (Figure 3).

At two years following his repair, a Doppler ultrasound demonstrated a sac measuring 82 (AP) x 94 (T) x 84 (L) mm compared to 173 (AP) x 156 (T) x 240 (L) mm at presentation. There was no evidence of a leak. Post-operative surveillance recommendations differ for open AAA repair compared to endovascular repair. For patients who undergo an open repair, the recommended surveillance intervals with color duplex ultrasound or CT is at 5, 10, and 15 years to evaluate for para-anastomotic aortic aneurysms. Those who have had an endovascular repair, however, require a 30-day and 6-month post-operative CT-angiogram and plain radiographs, followed by yearly Doppler ultrasound if body habitus permits adequate assessment or CT [9].

## Discussion

Inflammatory AAAs (IAAAs) were first described in 1972 and are currently defined by having extensive peri-aneurysmal fibrosis, very thickened aneurysmal wall, and extensive fibrous adhesions to adjoining tissue and structures [10,11]. These fibrous adhesions can make open repair technically difficult and may involve retroperitoneal structures including the duodenum or ureters. A feature of IAAAs is the mantle sign, defined as hypoechoic wall thickening of the anterior or anterolateral side of an aortic aneurysm [12]. IAAAs account for 3-10% of all AAAs and classically present with the triad of abdominal, flank, or back pain, weight loss, and an elevated erythrocyte sedimentation rate (ESR) [13]. This patient had an elevated C-reactive protein of 266 mg/L and ESR of 32 mm/h. 

The pathophysiology and events leading to the development of an IAAA is not fully understood. The development of non-inflammatory AAA is multifactorial and is associated with both environmental factors as well as a genetic predisposition. An inflammatory cell infiltrate into the aortic wall is common to both types but more pronounced in the adventitia of IAAAs [11]. More research is required to identify the factors involved in the formation of the dense fibrous tissue that is unique to IAAAs, which could in the future be a target in the management of IAAAs. 

One group of environmental factors that has been researched is the role of infection as the trigger for the inflammatory response. Infections that have been found to be associated with AAA and more prevalent in IAAAs are herpes simplex virus and cytomegalovirus [14]. Unfortunately, neither were tested for in this case.

The management of IAAAs may be non-operative or operative. Medical management could be considered in cases where the extent of fibrosis poses a technical challenge for operative management or the patient is not a surgical candidate. It includes anti-inflammatory and immunosuppression with steroids [11]. Surgical repair, however, remains the definitive treatment option. Further studies are required to determine if there is an advantage of open versus endovascular repair. Endovascular repair, however, has been reported as having reduced or no regression in the level of fibrosis and in one case showed the stent provoked an inflammatory response in a non-IAAA [15,16]. Patients with IAAA are at an increased risk of requiring further surgery for pseudoaneurysm repair. Prifti et al. [17] recently reported elevated ESR, ischemic heart disease, and chronic renal failure as strong predictors of poor overall outcome in patients with IAAA.

## Conclusions

To our knowledge, we report the largest non-ruptured IAAA in Queensland that required urgent retrieval from a rural hospital. This case highlights the importance of early detection and surveillance of AAAs as well as the management of risk factors by primary health care physicians. Australia does not have a population screening program for AAAs like in the United Kingdom or Sweden; however, primary care physicians are able to provide targeted screening of patients on the basis of risk factors such as family history of AAA, smoking history, and age. It is because of surveillance programs that presentations of such large AAAs are becoming less common.
